# Large scale interaction analysis predicts that the *Gerbera hybrida *floral E function is provided both by general and specialized proteins

**DOI:** 10.1186/1471-2229-10-129

**Published:** 2010-06-25

**Authors:** Satu Ruokolainen, Yan Peng Ng, Victor A Albert, Paula Elomaa, Teemu H Teeri

**Affiliations:** 1 Gerbera Laboratory, Department of Applied Biology P.O. Box 27 (Latokartanonkaari 7), FIN - 00014 University of Helsinki, Finland; 2 Current Address: Biomedicum Helsinki, P.O. Box 63 (Haartmaninkatu 8), FIN-00014 University of Helsinki, Finland; 3 Department of Biological Sciences, University at Buffalo (SUNY), Buffalo, NY 14260, USA

## Abstract

**Background:**

The ornamental plant *Gerbera hybrida *bears complex inflorescences with morphologically distinct floral morphs that are specific to the sunflower family Asteraceae. We have previously characterized several MADS box genes that regulate floral development in Gerbera. To study further their behavior in higher order complex formation according to the quartet model, we performed yeast two- and three-hybrid analysis with fourteen Gerbera MADS domain proteins to analyze their protein-protein interaction potential.

**Results:**

The exhaustive pairwise interaction analysis showed significant differences in the interaction capacity of different Gerbera MADS domain proteins compared to other model plants. Of particular interest in these assays was the behavior of SEP-like proteins, known as GRCDs in Gerbera. The previously described GRCD1 and GRCD2 proteins, which are specific regulators involved in stamen and carpel development, respectively, showed very limited pairwise interactions, whereas the related GRCD4 and GRCD5 factors displayed hub-like positions in the interaction map. We propose GRCD4 and GRCD5 to provide a redundant and general E function in Gerbera, comparable to the SEP proteins in Arabidopsis. Based on the pairwise interaction data, combinations of MADS domain proteins were further subjected to yeast three-hybrid assays. Gerbera B function proteins showed active behavior in ternary complexes. All Gerbera SEP-like proteins with the exception of GRCD1 were excellent partners for B function proteins, further implicating the unique role of GRCD1 as a whorl- and flower-type specific C function partner.

**Conclusions:**

Gerbera MADS domain proteins exhibit both conserved and derived behavior in higher order protein complex formation. This protein-protein interaction data can be used to classify and compare Gerbera MADS domain proteins to those of Arabidopsis and Petunia. Combined with our reverse genetic studies of Gerbera, these results reinforce the roles of different genes in the floral development of Gerbera. Building up the elaborate capitulum of Gerbera calls for modifications and added complexity in MADS domain protein behavior compared to the more simple flowers of, e.g., Arabidopsis.

## Background

Reproductive roles of MADS box genes in plants extend from determination of floral organ identity to other floral processes such as control of meristem identity and determinacy, inflorescence architecture, and induction or inhibition of flowering (reviewed in [1]). The current view is that MADS domain regulatory proteins accomplish this multitude of tasks by forming higher order complexes, which then act on promoter sequences of their target genes [2,3]. In the simplest model, the higher order complexes are tetramers, or 'dimers of dimers' [4,5]. In this 'floral quartet' model, sepal identity is defined by an AAEE quartet, petals by ABBE, stamens by BBCE and carpels by a CCEE quartet, the letters referring to functions of the MADS domain proteins in the ABC and the extended ABCDE models [4,6,7]. Other reproductive functions such as meristem identity could be controlled by similar tetramerous complexes in which at least the A and E function proteins are thought to participate [8]. However, none of these higher order complexes have been observed or verified *in planta*, and they could be larger and more extensive than tetramers of MADS domain proteins.

MADS transcription factors are highly conserved across the plant kingdom and are easily recognizable by the eponymous MADS domain, named after the first identified members of the gene family [9-13]. The general structure of the best studied type II MADS domain proteins consists of the conserved MADS and the plant-specific K (keratin-like) domains, which flank the less conserved I (intervening) domain, and the C (carboxy terminal) domains. All of these protein domains have been shown to be able to participate in dimerization processes. The MADS domain has further DNA binding capacity [14], whereas the variable C domain of some, but not all, MADS domain proteins contains amino acids that function in transcriptional activation [2,15].

The first observed MADS protein dimer was the B function heterodimer between DEFICIENS and GLOBOSA in *Antirrhinum majus *[13,16,17]. Many of the protein-protein interactions defined since then are highly conserved among homologs in both monocot and dicot plants [18]. The multimeric protein complexes interact with their target promoter sequences [2,3] by binding to *cis *elements with the canonical sequence CC(A/T_6_)GG, termed the CArG box [10,13,14,16,19-21]. Each MADS protein can participate in a number of different complexes, making the potential number of combinations, and thus target gene sets, very large. Most of the reported protein-protein interactions are between MADS domain proteins themselves, but involvement of other proteins have also been observed. Examples are the anther-specific secreted protein ATA20, the leucine zipper protein MIP1, the seed specific histone fold protein NF-YB, LEUNIG, which shares sequence similarity with yeast Tup1 corepressor, the plant specific regulatory protein SEUSS, and proteins PFMAGO1 and PFMAGO2, which are homologous to highly conserved RNA binding proteins involved in many developmental processes [3,22-26]. MADS domain protein complexes have also been shown to act on their own promoters to regulate their own expression, and to form autoregulatory loops that stabilize their expression after induction [16,25,27-36].

We have contributed to floral developmental genetics by investigating a model member of the sunflower family, the ornamental plant *Gerbera hybrida *(reviewed in [37]). The highly compressed inflorescences (capitula) of the Asteraceae family differ from other model systems in that they bear flowers of dissimilar type, showing differences in sexuality, morphology and sometimes coloration. The different flower types combine in the flower head into a second-order structure resembling a single large flower - an apparent pollination adaptation [17,38]. Control of flower and inflorescence development therefore has extra tiers in Gerbera. Not only must the correct floral organs develop in correct places, but also particular types of flowers must emerge along precise radial coordinates of the inflorescence. We have previously shown that many general principles of flower development apply to Gerbera [37,39,40], and that functional homologues for B, C and E function genes can be identified. However, Gerbera also has its own unique features. Whereas the *Arabidopsis thaliana SEPALLATA *(*SEP*) genes encode the E function in a redundant and whorl non-specific manner, among several Gerbera *SEP*-like MADS box genes, a paralogous pair (*GRCD1 *and *GRCD2*) has apparently undergone subfunctionalization, showing non-redundant whorl-specific functions in stamen and carpel development, respectively [41,42]. Interestingly, Gerbera MADS box genes also show differential expression patterns along the radius of the capitulum, suggesting that different complexes may act on flower primordia to engender their different developmental fates [43].

Studies on MADS domain protein higher-order complexes have been carried out in Arabidopsis, snapdragon, *Petunia hybrida *and tomato [2,3,23,44-46]. Our aim was to map MADS domain protein-protein interactions in Gerbera, and to compare these interactions between Gerbera and other model systems. In this study, a total of fourteen Gerbera MADS domain proteins active (or suspected to be active) in reproductive development were included in an interaction study using yeast two- and three-hybrid assays. These data, in combination with our previous reverse genetics studies, provide intriguing new information for Gerbera MADS domain proteins. Along with the highly specialized E function proteins GRCD1 and GRCD2, Gerbera harbors a redundant pair of E function proteins, GRCD4 and GRCD5, which have an apparently general non-whorl-specific function. Despite functioning as obligate heterodimers [13,19], B function proteins of Gerbera are able to participate in higher order complexes as independent proteins. Furthermore, they have an extensive interaction capacity when present as a dimer. The B function proteins show interaction with C function proteins and with all Gerbera SEP-like proteins except with staminodia-determining GRCD1. This might indicate a special role for GRCD1 as a whorl- and flower-type specific C function partner.

## Results

### Phylogenetic positioning of Gerbera MADS box genes

Of the tested Gerbera MADS box genes, *GSQUA1*, *GGLO1*, *GDEF1*, *GDEF2*, *GAGA1*, *GAGA2*, *GRCD1*, *GRCD2 *and *GRCD3 *were included in a phylogenetic tree published previously [41] and were placed among orthologous genes from other plant species. The phylogenetic placements of *GRCD1 *and *GRCD2 *was further refined by Zahn *et al*. [47], who showed them to be more distantly related paralogs than previously suspected. *GRCD4 *and *GRCD5 *(and the above genes) were added to the data set of Zahn *et al. *[47] and phylogenetic results show them to be related to other *SEP*-like genes (Additional file 1, Figure S1). The phylogenetic position of Gerbera *SQUAMOSA/APETALA1 (SQUA/AP1) *-like genes (*GSQUAs*) is reported elsewhere [48]. To summarize, *GSQUA1 *and *GSQUA3 *group together with *AP1 *and *CAULIFLOWER *(*CAL*) of Arabidopsis [49,50], while *GSQUA2*, *GSQUA4*, *GSQUA5 *and *GSQUA6 *are phylogenetically closer to the Arabidopsis *FRUITFULL *(*FUL*) gene [51,52].

### Expression patterns of Gerbera *SEP*-like genes

Of eudicot MADS box genes, C and B function genes generally show a narrow expression pattern, which directly reflects their function in carpel, stamen, and petal development, respectively [27,53,54]. On the other hand, the *Arabidopsis SEP *genes, necessary for several processes in floral development, are widely expressed in flowers [55-57]. In order to gain potential insight into their function and interaction range, the expression patterns for Gerbera MADS box genes were studied using RNA gel blots and *in situ *hybridization.

The expression patterns for *GSQUA1, GDEF1, GDEF2, GGLO1, GAGA1, GAGA2, GRCD1, GRCD2, GSQUA2, GSQUA3*, and *GSQUA5 *were reported previously [39,41,42,48] (See Additional file 2, Table S1).

**Figure 1:**
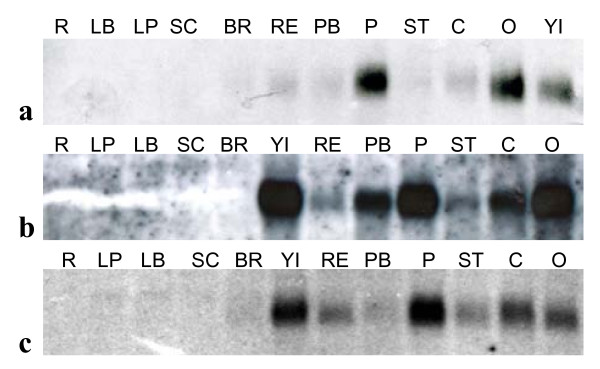
**Expression patterns of (a) *GRCD3*, (b) *GRCD4*, and (c) *GRCD5 *in different Gerbera organs by RNA gel blots. R, roots; LP, leaf petiole; LB, leaf blade; SC, scape; BR, bracts; RE, receptacle; PB, pappus bristles; P, petals; ST, stamens; C, carpel; O, ovary; YI, young inflorescence (6-16 mm in diameter)**. All *GRCD*s studied here display similar, widespread inflorescence abundant expression pattern.

According to RNA gel blots probed with a gene specific probe, strongest expression of *GRCD3 *was seen in inflorescence, petals and ovary. GRCD3 was also expressed in carpel and receptacle and weak expression was detectable in stamens, pappus bristles and bracts (Figure 1a). *GRCD3 *expression was found to be strongest during the earlier stages (1-7; see [58]) of Gerbera ray flower petal development (Additional file 3, Figure S2), and only very weak expression was seen at the last stages assayed, 10-11.

*GRCD4 *expression was found to be strongest in young Gerbera inflorescences (6-16 mm in diameter) and in petals and ovaries, while carpels and pappus bristles gave a slightly weaker signal for expression in the RNA gel blot. Weak, but detectable signal was observed in the receptacle and stamens. No signal was detected in the inflorescence-derived green organs (scape and bracts), or vegetative organs, which included leaf petioles, leaf blades and roots (Figure 1b). *GRCD4 *was expressed throughout ray flower petal development, clearly fading toward later developmental stages (Additional file 3, Figure S2).

The expression of *GRCD5 *was inflorescence-abundant according to an RNA gel blot probed with a gene specific probe. *GRCD5 *was expressed in all floral whorls, with the strongest expression detected in young inflorescence (6-16 mm) and petal samples. Slightly weaker expression was detected in receptacle, stamens, carpel and ovary. In bracts and the outmost floral whorl of Gerbera, pappus bristles, *GRCD5 *was expressed at a very low level (Figure 1c). Interestingly, the expression of *GRCD5 *differs from the expression of * GRCD3 *and *GRCD4 *during ray flower petal development. Both *GRCD3 *and *GRCD4 *were expressed at earlier stages of development, whereas *GRCD5 *showed remarkable upregulation in the late stages, when the Gerbera inflorescence starts to open (Additional file 3, Figure S2). Our microarray study supported this observation [59].

**Figure 2:**
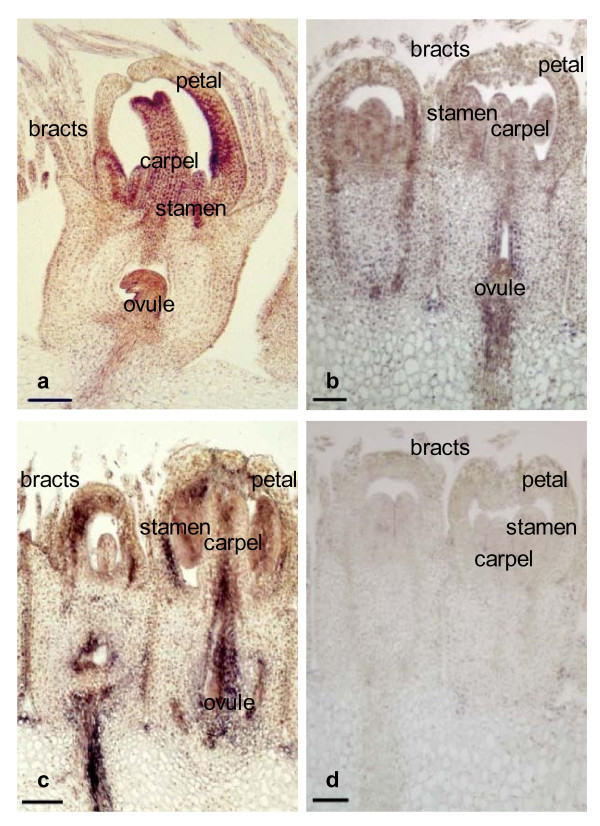
**Expression of Gerbera *GRCD3*, *GRCD4*, and *GRCD5 *at *in situ *level**. (a) *GRCD3 *anti-sense 13 mm, (b) *GRCD4 *anti-sense 14 mm, (c) *GRCD5 *anti-sense 12.7 mm, (d) *GGLO1 *sense 14 mm as negative control. Size of the inflorescence diameter given in mm.

*GRCD3 *was expressed in several floral organs as shown by *in situ *hybridization. Strong expression was visible in ovule, carpel and petals. Slightly weaker expression was discovered in stamens and pappus bristles (Figure 2a). *In situ*, both *GRCD4 *and *GRCD5 *were widely expressed in all floral whorls, confirming the results shown by RNA gel blots (Figure 2b and 2c). Overall, the expression patterns for both *GRCD4 *and *GRCD5 *were remarkably similar.

### Gerbera SEP-like proteins are involved in broad pairwise interactions

Pairwise interaction capacity between Gerbera MADS domain proteins was tested for all combinations of the fourteen proteins using the yeast two-hybrid assay. The Gerbera proteins were translationally fused separately to both the binding domain and the activation domain, and were combined in both directions.

**Figure 3:**
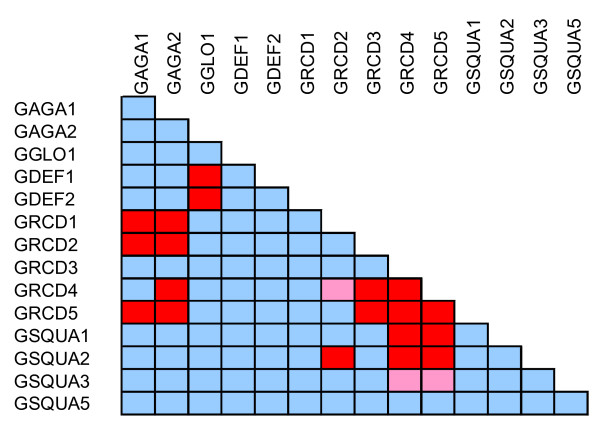
**Yeast two-hybrid analysis of protein-protein interactions among Gerbera MADS domain proteins**. Red, strong interaction; pink, weak interaction; blue, no interaction detected.

As summarized in Figure 3, the most broadly interacting proteins in pairwise assays are those phylogenetically grouping with the E function, or SEP-like, MADS domain factors. GRCD4 and GRCD5 each interacted with eight of the fourteen proteins in the interaction screen, including the only two self-interactions (homodimer formation) observed among our assays. These factors interacted with all other Gerbera MADS domain proteins except those from the B-clade (i.e., GGLO1, GDEF1 and GDEF2), the SEP-like protein GRCD1, and the FUL-like protein GSQUA5. In addition, GRCD4 did not interact with GAGA2, and GRCD5 not with GRCD2. Both GRCD4 and GRCD5 were assayed as truncated proteins due to a strong autoactivation reaction.

GRCD1 and GRCD2 have been functionally characterized previously, and they take part in whorl-specific homeotic functions in stamens and carpels, respectively. Furthermore, GRCD2 is required for meristem identity and determinacy [41,42]. Compared to the Arabidopsis SEP proteins, these two Gerbera proteins showed a limited interaction capacity in our assays. GRCD1 and GRCD2 both interacted with the C-function proteins GAGA1 and GAGA2, as we have previously observed. In addition, GRCD1 had no other pairwise partners, while GRCD2 interacted with GRCD4 and GSQUA2. GRCD3 has perhaps the most limited interaction pattern of the E-class family, interacting pairwise only with GRCD4 and GRCD5. GRCD3 is placed in the outermost branch together with AGL6 of Arabidopsis [60].

The floral homeotic C function genes *GAGA1 *and *GAGA2 *show similar expression patterns and similar transgenic phenotypes [39]. Indeed, GAGA1 and GAGA2 behaved similarly as well in protein interaction assays, forming dimers with the SEP-like Gerbera proteins GRCD1, GRCD2 and GRCD5. GAGA2 also formed a dimer with GRCD4.

The homeotic B function is represented in Gerbera by the genes *GGLO1 *and *GDEF2*. These genes show strong whorl-specific expression patterns typical of B function MADS box genes, as well as characteristic homeotic changes in transgenic Gerbera lines [39,61]. Gerbera also harbors a *TM6*-like gene, *GDEF1*, which is closely related to *GDEF2*, but based on its expression pattern and transgenic analyses, apparently does not contribute to the classical B function. Recent results indicate that *TM6-*like genes take part in the control of stamen development [62,63], also in Gerbera [61]. The GGLO1 and GDEF2 proteins show strong interaction as expected for a pair responsible for the B function. Interestingly, GDEF1 also interacts with GGLO1 in yeast. In a pairwise interaction assay, these three proteins do not interact with any other Gerbera MADS domain proteins.

Homeotic A function genes have not been described in Gerbera - in fact MADS box genes responsible for sepal and petal identity as per the ABC model have not been identified in plants other than Arabidopsis, (reviewed in [40,64]). Nevertheless, Gerbera contains several genes similar to the Arabidopsis A function MADS box gene *AP1 *and its paralogs *CAL *and *FUL*, or its ortholog in snapdragon, *SQUA *[49-52,65]. Altogether six *SQUA*-like genes have been identified in Gerbera [39,59,48]. Full length cDNAs for *GSQUA1*, *GSQUA2*, *GSQUA3 *and *GSQUA5 *were included in this study. The corresponding proteins did not interact among themselves in any pairwise combination. All but GSQUA5 interacted with the SEP-like protein GRCD5. Interestingly, GSQUA2 also interacted with GRCD2 in the pairwise assay. GRCD2 represents another SEP-like gene in Gerbera, with pleiotropic functions in carpel identity, floral meristem identity and inflorescence determinacy [42].

### Higher order complexes between Gerbera MADS domain proteins

Plant MADS domain proteins are known to bind DNA only after dimerization [16,19]. However, their function in the regulation of flower development has been implied to involve formation of higher order protein complexes, possibly tetramers as depicted in the floral quartet model [4,7]. We tested if higher order complex formation could be promoted between Gerbera MADS domain proteins that did not show pairwise interactions. This was done by introducing a third protein into the system in the yeast three-hybrid assay. For example, SEP-like proteins have previously been reported to act as "glue proteins" by facilitating interactions between partners that remain inactive in yeast two-hybrid studies [3,46]. In the yeast three-hybrid assay, care was taken to avoid a positive signal due to a pairwise interaction. However, the assay became uninformative for this reason only in situations where the three proteins all interacted pairwise. Our survey was not exhaustive, but out of 531 possible (informative) combinations, 313 that were considered to be of high relevance were tested. In a few cases, we found out that an activation function emerged when two MADS domain proteins interacted. This type of autoactivation (see below) was unexpected but is interesting. In practice, however, it resulted in some uninformative three-hybrid assays.

**Figure 4:**
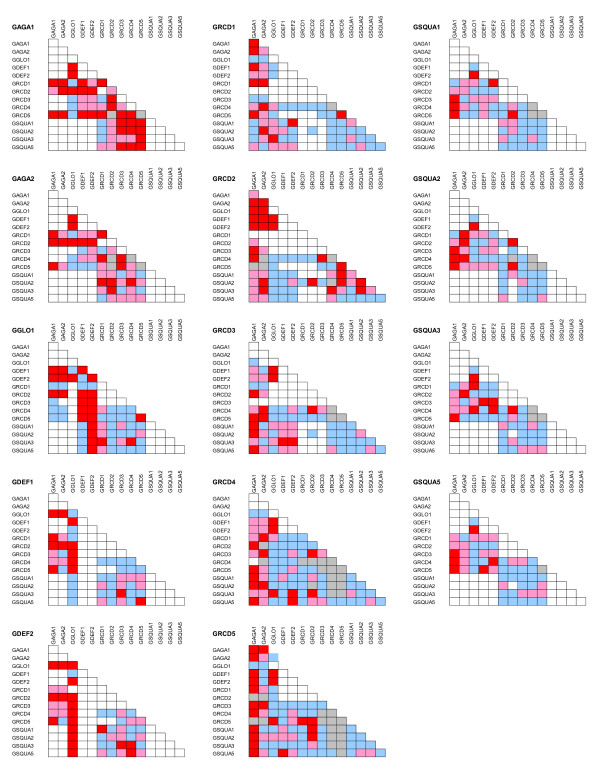
**Yeast three-hybrid analysis of ternary protein complex formation among Gerbera MADS domain proteins**. Red, strong interaction; pink, weak interaction; blue, no interaction; grey, interaction test uninformative; white, not tested.

The Gerbera B function proteins GGLO1 and GDEF2 formed a closed interaction pair showing pairwise association only with each other. Still, the GGLO1/GDEF2 heterodimer is involved in different developmental processes, leading to petal development in whorl 2 and stamen development in whorl 3. We tested formation of higher order complexes by fusing GDEF2 (or GDEF1) to the binding domain in pDEST32, then providing GGLO1 as an unfused protein in pARC351 to the yeast cells, and assaying which Gerbera MADS domain proteins (those not interacting with GDEF1 or GDEF2), fused with the activation domain of pDEST22, gave a positive signal when GGLO1 was already complexed with either GDEF1 or GDEF2. In these assays, both C-function proteins GAGA1 and GAGA2 interacted with the GGLO1/GDEF1 and GGLO1/GDEF2 dimers. All Gerbera SEP-like proteins except GRCD1 showed interaction with GGLO1/GDEF1 and GGLO1/GDEF2 dimers as well, although none of them interacted with the B-class proteins alone. Similarly, all GSQUA proteins interacted with the dimer GGLO1/GDEF2 - but none of them with the dimer GGLO1/GDEF1 (Figure 4).

These results indicate that GGLO1, GDEF1 and GDEF2 are activated not only for their (putative) transcriptional function by heterodimer formation, but also for their capacity to recruit additional proteins to form a transcriptional complex. We also observed that all three B clade proteins were, individually, captured into higher order complexes when expressed together with various combinations of GRCD and GSQUA proteins. Further, combinations of a GAGA protein and a GRCD protein typically recruited the GDEF proteins, but not GGLO1, in higher order complexes (Figure 4).

The two C-clade genes GAGA1 and GAGA2 have been considered similar in their function based on expression patterns and transgenic analysis. Also the pairwise interaction patterns of GAGA1 and GAGA2 are very similar. The test for higher order complexes with yeast three-hybrid assay showed differences, however. Generally GAGA1 was more active in threeway complexes than GAGA2, and in addition they showed complementary specificities in some cases. GAGA2 was active together with GRCD1 and GSQUA proteins while GAGA1 was not, and GAGA1 was active with several other GRCD/GSQUA combinations where GAGA2 was not active.

### Emerging transcriptional activation by complex formation

Interestingly, when the dimers GGLO1/GDEF1 and GGLO1/GDEF2 were combined with an empty activation domain containing vector pDEST22, or completely without an activation domain carrying plasmid, yeast growth resulted on plates selecting for weaker interactions (Additional file 4, Table S2). This was unexpected, since transcriptional activation of MADS domain protein complexes are thought to be brought on by specific members of the complex, typically proteins of the SEP family [66]. In order to avoid false results from the yeast three-hybrid assay, we reassayed all sets of binding domain/unfused proteins where yeast growth was observed, irrespective of which MADS domain protein was fused to the activation domain (Additional file 4, Table S2). In addition to growth under weak selection for the GGLO1/GDEF1 and GGLO1/GDEF2 dimers mentioned above, we observed that the combinations GAGA1/GRCD2, GAGA2/GRCD2 and GSQUA2/GRCD2 (without added activation domain) resulted in prominent growth of yeast under strong selection. All of these proteins interacted pairwise in yeast two-hybrid assay, but none of them alone had autoactivation capacity. While obstructing a number of yeast three-hybrid results (see Additional file 4, Table S2), this phenomenon is interesting in itself and shows that transcriptional activation may be a combined function of two interacting proteins.

## Discussion

The major aim of this work was to investigate the interaction capacity of Gerbera MADS domain proteins using the yeast two-hybrid and three-hybrid assays. In addition, we report expression patterns for three Gerbera *SEP*-like MADS box genes, *GRCD3*, *GRCD4*, and *GRCD5*. Expression of other Gerbera MADS-box genes has been published previously (see Additional file 2, Table S1). Although protein interaction in yeast is not always conclusive for interaction *in planta*, our analysis uncovered intriguing information that can be used to compare and classify Gerbera MADS domain proteins with reference to those of Arabidopsis and Petunia, as well as to speculate, in the light of our reverse genetics studies, about the regulatory roles of MADS box genes during differential development of flowers and floral organs in Asteraceae.

### *GRCD4 *and *GRCD5 *encode general E function proteins in Gerbera

E function proteins have been reported to be particularly active in forming heterodimers in yeast two-hybrid experiments in several plant species [8,18,23,44,67]. For example, the Arabidopsis proteins SEP1 and SEP3 form nodes in the pairwise interaction map and are thought to be responsible for transcriptional activation of a number of different higher order complexes of MADS domain proteins [67]. At least one of the redundant SEP proteins is required for floral organ identity determination where specificity is determined through the combination of A, B and C function proteins [3]. In Gerbera, similarly to the (redundantly encoded) E function in Arabidopsis, proteins from the SEP clade are needed to accomplish correct organ identity determination, and specifically, to mediate the activity of the Gerbera C function encoded by the genes *GAGA1 *and *GAGA2 *[41,42]. As the *SEP*-like Gerbera gene *GRCD1 *is needed for stamen (more precisely, staminode) identity determination, and *GRCD2 *for carpel identity determination, we have concluded that the homeotic E function has evolved into a whorl-specific set of subfunctionalized gene paralogs [41,42].

Mapping of Gerbera MADS domain protein interactions sheds new light on these conclusions and to the roles of Gerbera *SEP*-like genes. While the Gerbera GRCD1 and GRCD2 proteins have evolved to carry out whorl specific functions similar to the E function in Arabidopsis, they have concomitantly lost their general focal position in the interaction map - they interact with a very limited number of other MADS domain proteins, in fact nearly exclusively with the C function GAGA proteins, for which they were described as necessary companions based on transgenic phenotypes [41,42].

**Figure 5:**
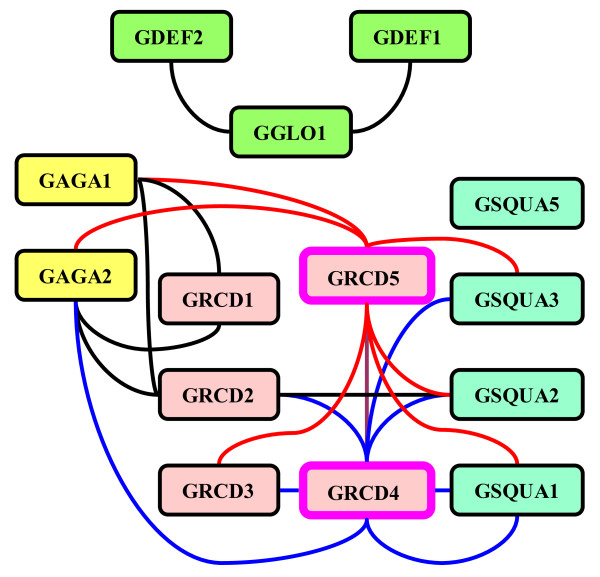
**Gerbera MADS domain protein pairwise interaction map**. The central interactions with SEP-like proteins GRCD4 and GRCD5 are shown in blue and red, respectively. Interaction between GRCD4 and GRCD5 is illustrated in purple. Interactions among other MADS domain proteins are shown in black. Proteins lined in pink, GRCD4, and GRCD5, were the only Gerbera MADS domain proteins which formed homodimers (not illustrated). The color coding shows SQUA-like proteins as light blue, SEP-like proteins as pink, B function proteins as green, and C function proteins as yellow rectangles.

Instead, GRCD4 and GRCD5 display a hub-like position in the Gerbera interaction map similar to SEP1 and SEP3 in Arabidopsis (Figure 5). Further, like SEP1 and SEP3, GRCD4 and GRCD5 harbor transcriptional activation domains based on their autoactivation capacity in yeast, making them the most likely Gerbera candidates for the necessary and general, whorl non-specific floral E function proteins. This is supported by their expression analysis, which shows that GRCD4 and GRCD5 are expressed in all floral whorls. Although their interaction capacity is partly complementary (Figure 3), lack of prominent transgenic phenotypes for either of them downregulated alone (data not shown) suggests redundancy. Specifically, this interpretation predicts that a double transformant (with both *GRCD4 *and *GRCD5 *downregulated) should show a strong (negative) floral phenotype.

### GRCD1 and GRCD2 provide specialized functions in Gerbera

While a general E function is encoded by *GRCD4 *and *GRCD5 *in Gerbera, our interaction data reinforces the conclusion [41,42] that *GRCD1 *and *GRCD2 *are specific and necessary activators of the Gerbera C function, encoded by the genes *GAGA1 *and *GAGA2*. However, the whorl specificity of *GRCD1 *and *GRCD2 *function remains incompletely characterized. Both genes are expressed in whorls three and four, and both proteins interact with GAGA1 and GAGA2. Nevertheless, *GRCD2 *cannot replace *GRCD1 *in whorl three, and *GRCD1 *cannot replace *GRCD2 *in whorl four [41,42]. Furthermore, transgenic Gerbera plants in which *GRCD1 *is downregulated carry petal-like organs in whorl three of ray flowers (in place of the staminodia in non-transgenic plants), but the stamens of the central disc flowers are nearly normal and male fertile. As we have concluded previously, redundant action by other MADS domain proteins may be taking over the function of GRCD1 in disc flowers [41]. Unless a very low level of expression for *GRCD1 *is sufficient for normal stamen development in disc flowers, we have yet to determine a disc-flower whorl-three-specific GRCD1-like C function activator in Gerbera.

### Differential interactions with the B clade proteins

Both the developmental and biochemical aspects of B function genes have been found to be highly conserved (reviewed in [68]). B function proteins are necessary for petal and stamen development [6]. As Gerbera flower types differ in size and development of exactly these two organs, the behavior of B function proteins is of particular interest to us. We have earlier concluded that arrest of stamen development in marginal flowers is not due to differential expression of *GGLO1 *or *GDEF2 *in developing flower primordia; both genes are expressed strongly at early stages of all flower types [39].

Yeast two-hybrid results indicated that both GDEF1 and GDEF2 proteins readily form heterodimers with GGLO1. Remarkably, the B function dimers were discovered to have an intrinsic activation capacity, not present in the proteins when expressed alone in yeast (Additional file 4, Table S2). It is well known that dimerization of MADS domain proteins is necessary for their capacity to bind their target sequences (CArG boxes) in DNA [16], but it has apparently gone unnoticed that dimerization may also render them functional in transcriptional activation, at least in yeast. Other proteins are expected to enhance activation by bringing in more activation capacity; however, even without external activation, the B protein dimer is active.

Yeast three-hybrid results further indicate that GGLO1, GDEF1 and GDEF2 are also activated by heterodimer formation for the capacity to recruit additional proteins in higher order complexes. Both GLO1/GDEF1 and GGLO1/GDEF2 heterodimers interact strongly with the Gerbera C function proteins GAGA1 and GAGA2. Similarly, all GSQUA proteins interact with the B function dimer GGLO1/GDEF2, but none of them with the GGLO1/GDEF1 dimer. The latter is the clearest difference we observed regarding the two Gerbera DEFICIENS-like proteins, and it demonstrates that GDEF1 and GDEF2 are not simply redundant. Instead of the classical B-function proposed for GDEF2, expression pattern and transgenic phenotypes for the *TM6*-like *GDEF1 *suggest a role in stamen development [61].

Although none of the Gerbera SEP-like proteins (GRCDs) interact pairwise with the B-clade proteins, all of them - except GRCD1 - show strong interaction with GGLO1/GDEF1 and GGLO1/GDEF2 complexes. This may speak for a very specific role for GRCD1 as a whorl- and flower-type specific C function partner. The observation relates to the findings of Ito *et al. *[69] regarding an early, traditional homeotic role of *AG *in flower organ development, and a late function in anther development. The latter requires postulation of a yet-undescribed activator of *AG *in Arabidopsis [69], providing yet another example where the C function is modulated in a certain developmental context.

### Single Gerbera B clade proteins participate in higher order protein complexes

Gerbera B function proteins also participate in trimerous complexes as single proteins. A recent study with tomato proteins showed similar results [45]. Although GDEF2 and GGLO1 are traditionally thought to form an obligatory heterodimer to conduct their joint function, and show dramatically increased interaction capacity when expressed together in yeast three-hybrid, they also show individual interactions with pairs of GAGA and GRCD proteins. The B function proteins alone are also capable of participating in trimerous complexes where none of the involved proteins interact pairwise. GGLO1 complexes with GRCD1 and GSQUA3, or with GRCD4 and GSQUA3, as well as with GRCD5 and GSQUA2. GDEF2 complexes with GSQUA1 or GSQUA5 when GRCD1 is present, and weakly with GSQUA2 or GSQUA5 when GRCD5 is present. It is not clear what roles, if any, lone B function proteins may have in floral development.

### The pleiotropic GRCD2 protein has hidden activation capacity

*GRCD2 *has an extended role in flower development and controls carpel identity, floral meristem identity and inflorescence determinacy [42]. Down regulation of *GRCD2 *in transgenic plants affects on all these processes, but ectopic expression of *GRCD2 *does not lead to observable phenotypes, indicating that this protein's activity is dependent on additional factors. We have previously observed (and verify here) protein-protein interactions between GRCD2 and both of the Gerbera C-function GAGA proteins, which is in concordance with both the homeotic and floral meristem identity role of GRCD2. To our surprise, GRCD2/GAGA1 and GRCD2/GAGA2 dimers showed strong capacity for transcriptional activation, not present for any of the proteins alone. Another protein pair which gains marked transcriptional activation upon dimerization is GRCD2/GSQUA2. G *SQUA2 *has, unlike any of the other MADS box genes of Gerbera, a strong flowering inducing capacity when expressed ectopically [48]. Both *GRCD2 *and *GSQUA2 *are co-expressed in the young undifferentiated inflorescences at the early stages of development, and later the expression patterns of these genes overlap in several floral organs [42,48].

### A multitude of higher order protein complexes may be critical for Gerbera stamen development

Based on the protein interactions presented in this study, we propose hypothetical higher order protein complexes involved in Gerbera stamen identity determination. For determination of stamen identity, a B function protein pair (GDEF2/GGLO1) is required, along with a C function protein (GAGA1 or GAGA2). Due to the expression pattern of *GDEF1*, and its links to stamen development [61], the GDEF1/GGLO1 dimer may also be involved. The broad interaction capacity and transcriptional activation properties of GRCD4 and GRCD5 suggest that they, or one of them, are needed for development of all floral organs by bringing together higher order protein complexes and activating them. In this scenario, an obvious deviation from the quartet model is that the number of MADS domain proteins required for stamen development exceeds four.

GRCD1 is needed for stamen development in marginal flowers, and in pairwise assays both Gerbera C function proteins interact with GRCD1. We postulate that even if GRCD1 does not interact directly with the B function protein pair (Figure 4), one possibility is that it would participate in a higher order protein complex by first forming a protein dimer with a GAGA protein. Alternatively, the weaker interaction capacity of GRCD1, compared to other GRCD proteins, could indicate a compromised function, easily competed out by other components necessary for stamen development and eventually leading to release of the developmental arrest in central flowers. Data from microarray experiments show that *GRCD1 *is in fact upregulated in marginal flowers compared to disc flowers [43].

*In vitro *data confirming a specific role for MADS domain protein tetramers has recently emerged [70,71], but *in planta *data is still lacking. Combined analysis of interaction between Gerbera MADS domain proteins indicate that the actual higher order protein transcriptional complexes could be larger than proposed by the quartet model, or (see also [72]) that higher order complex formation in quartets could be transient, with different proteins participating in an alternating manner. Relatively broad expression patterns, especially of *GRCD1-5 *[[41,42] and this paper] and *GSQUA2-5 *[48], summarized in Additional file 5, Table S3, provide opportunities for both types of increased complexity.

## Conclusions

Our study shows that Gerbera MADS domain proteins are capable of forming a multitude of higher order complexes in yeast assays. Gerbera MADS domain protein behavior in higher order complexes displays both characteristics that are common to all higher eudicots, but also specialized features, some of which may be specific to Asteraceae and its complex inflorescence structure. For example, in Gerbera the E function is split between the highly specialized GRCD1 and GRCD2 factors, which are active in stamens and carpels, respectively [41,42], and a more general activation capacity provided by GRCD4 and GRCD5. In other model species, such division of labor among E function proteins has not been observed to this extent. However, petunia E class proteins also differ in their higher order complex formation capacity, and single mutant analysis shows only minor phenotypic changes [73,74,62]. In contrast to what has been observed for B function proteins in general, Gerbera B function factors (GGLO1, GDEF2 and GDEF1) can participate in higher order complexes as single proteins, with the requirement for heterodimerization bypassed. Based on the data presented here, we speculate that the differential development of Gerbera flower types, especially that of the stamen whorl, requires more complexity than development of flowers in simple inflorescences that bear uniform flowers.

## Methods

### Gerbera MADS box genes used in interaction studies

Isolation of Gerbera MADS box genes *GGLO1, GDEF1, GDEF2, GAGA1, GAGA2 *and *GSQUA1 *has been reported previously [39]. Isolation of the Gerbera *AP1/SQUA*-like genes (*GSQUAs*) is described elsewhere [48]. *GRCD3 *was cloned from a petal library using a degenerate oligonucleotide [75] encoding an eight amino acid sequence of Arabidopsis AGAMOUS. *GRCD4 *and *GRCD5 *were identified as full-length cDNA clones from the Gerbera EST collection [59]. The recently identified paralogue of GDEF2, *GDEF3 *[61], was not included in this study. Summary of Gerbera MADS box genes used in this study is shown in Additional file 2, Table S1.

### Phylogenetic analysis

Parsimony analyses were performed on a nucleotide sequence matrix, modified from [47] to include all Gerbera *GRCD *genes. The modified data set was first translated to aid alignment, and then precisely back-translated to yield the original DNA sequences. The data set was analyzed using the TNT application [76] with the "new technology" option in a driven search using sectorial searches, tree-drifting and tree-fusing [77]. Analyses were run until a stabilized consensus had occurred twice using equal character weights and tree bisection-reconnection (TBR) branch swapping. Additional TBR branch swapping was performed on trees resulting from the initial search to find additional equally parsimonious trees. Bootstrap support for internal branches was also estimated using TNT. The majority rule consensus tree is shown collapsed for all branches with less than or equal to 50% bootstrap support. Two hundred and fifty replicates were conducted, each performing TBR branch swapping with 10 random entry orders saving one tree per replicate. Absolute support values are reported.

### Expression analysis of *GRCD3*, *GRCD4 *and *GRCD5*

Total RNA from different plant organs and from different developmental stages of petals (stages 1-11, according to [58]) was isolated using Trizol reagent (Invitrogen, cat. no. 11596-018). Equal amounts (10 μg) of RNA were run in a 0.8% agarose gel as described in [58]. The rRNA bands were visualized by EtBr staining to record even loading of the gel. The RNA was blotted on a membrane (Hybond-N, Amersham Biosciences) and hybridized (UltraHyb hybridization buffer, Ambion) with a gene-specific probes (213, 245 and 314 bp) designed from the 3' ends of *GRCD3, GRCD4 *and *GRCD5 *cDNA molecules, respectively. Probes were labeled with [^32^P] dCTP and hybridized at +42°C 16 h. The membranes were washed with 1 × SSC, 0.1% SDS at +42°C for 20 minutes. Subsequent washes were performed at +65°C for 15 minutes, 1-2 times.

*In situ *hybridization analysis was performed as described in [78,79]. *GRCD3, GRCD4 *and *GRCD5 *gene-specific sense and antisense probes (213, 245 and 314 bp) were prepared and quantitated using DIG RNA labeling kit (Boehringer Mannheim) according to the manufacturer's instructions. 10 μm thick paraffin sections were mounted in 50% glycerol after hybridization.

### Construction of Gateway entry plasmids

All full length Gerbera MADS box genes were introduced as cDNAs into the Gateway system using PCR (PCR Cloning System with Gateway Technology with pDONR221, Invitrogen). Primers flanking the first methionine of the gene and the stop codon were designed according to Invitrogen's instructions. Two nucleotides were added between the *attB1 *sequence and the start codon. Primers and Gateway sequences are shown in Additional file 6, Table S4.

The PCR products were purified and recombined with pDONR221 (Invitrogen) plasmid to create Gateway entry clones according to the manufacturer's instructions. All entry clones were sequenced to eliminate possible PCR artifacts.

### Yeast assays

Entry plasmids carrying Gerbera MADS box genes were recombined with the activation and binding domain containing plasmids pDEST22 and pDEST32 (Invitrogen) and transformed to yeast (*Saccharomyces cerevisiae*) strains PJ69-4A and PJ69-4α [80]. All plasmids were introduced in both yeast mating types. The pDEST32 clones containing N-terminal binding domain fusions were tested for autoactivation by plating them on the yeast medium SD (0.67% yeast nitrogen base without amino acids, 2% glucose, and appropriate amino acids) lacking adenine (SD -Ade), or histidine (SD -His) and supplemented with 1, 5 or 10 mM 3-amino-1,2,4-triazole (3-AT) (Sigma A8056). Autoactivation was observed for GRCD4 and GRCD5, and C terminal deletions were introduced to these constructs [81]. Deletions (Additional file 7, Figure S3) were designed so that the predicted alpha helical structure that starts within the conserved K domain and extends towards the C terminus of the protein was retained [82]. After deletions, autoactivation of the truncated constructs were retested both in absence and presence of an empty activation domain containing vector and were found negative. To obtain yeast double transformants, the A and α types of yeast strains were mated by pipetting them on top of each other on rich medium (SD Glu Complete). Yeast double transformants were plated on selection plates SD -Leu -Trp -Ade and SD -Leu -Trp -His + 1, 5 or 10 mM 3-AT. The plates were incubated at +22°C for 5 days. We scored a positive signal for interaction capacity if either of the directions resulted in growth of yeast on the selection medium (Additional file 8, Figure S4). Reciprocal tests gave the same result in all cases, except that the truncated GRCD4 fused to the binding domain gave consistently poor growth with other Gerbera MADS domain proteins except GRCD4 (homodimer formation) and GRCD5 (Additional file 9, Table S5).

For yeast three-hybrid assays, the plasmid pARC351 (Gateway compatible pRED-NLSa plasmid derivative, P. Ouwerkerk; Gateway modifications by R. Immink) was used to express the third protein of interest in yeast cells. The previously cloned Gateway entry plasmids were recombined with pARC351 according to Invitrogen's instructions. The purpose of this assay was to see whether two proteins inactive in yeast two-hybrid experiment could interact in the presence of a third protein. 313 combinations of three proteins were tested (Additional file 4, Table S2). The criteria for selecting the combinations was decided based on the ABC(DE) model and the previous results [2,6,7,83,73,85,45] The yeast three-hybrid interactions were selected on plates SD -Leu -Trp -Ura -Ade and SD -Leu -Trp -Ura -His, with 1, 5 and 10 mM, or 10 and 25 mM 3-AT, respectively. The plates were incubated at +22°C for 7 days.

Some yeast three-hybrid combinations gave positive signals regardless of which protein was fused with the activation domain. For these combinations we run extra controls where the activation domain containing vector pDEST22 was left out or was present empty in the yeast cells. In these controls, strong intrinsic autoactivation was discovered for Gerbera MADS domain protein dimers GRCD2/GAGA1, GRCD2/GAGA2, and GRCD2/GSQUA2, rendering some of the studied ternary protein complexes uninformative (see Additional file 4, Table S2). The limitation applied to protein combinations where GRCD2 was fused to the binding domain containing vector pDEST32, and GAGA1, GAGA2, or GSQUA2 was supplied from pARC351 vector, or *vice versa*. Gerbera B-clade protein dimers GGLO1/GDEF2 and GGLO1/GDEF1 selected under milder conditions exhibited similar autoactivation, but ternary complexes containing either Gerbera B-clade protein dimer and activating under stringent selection were scored as true positives.

## Authors' contributions

SR participated in the design of the experiment, carried out the expression analyses and the interaction experiments and drafted the manuscript. YPN participated in setting up the Gateway system. VAA performed the phylogenetic analysis, participated in the analysis of the results and helped to draft the manuscript. PE participated in the design of the experiment, analysis of the results and helped to draft the manuscript. THT coordinated the research, participated in the design of the experiment and analysis of the results and helped to draft the manuscript. All authors read and approved the final manuscript.

## Supplementary Material

Additional file 1 **Phylogenetic tree**. A phylogenetic tree showing the positions of Gerbera *SEP*-like genes. Click here for file

Additional file 2 **Gerbera MADS box genes**. Summary of Gerbera MADS box genes used in this study. Click here for file

Additional file 3 **Expression during ray flower development**. RNA gel blots showing expression of *GRCD3*, *GRCD4 *and *GRCD5 *during Gerbera ray flower development. Click here for file

Additional file 4 **Original yeast three-hybrid data**. Scores and interpretation of all yeast three-hybrid data collected. Click here for file

Additional file 5 **Expression of Gerbera MADS box genes**. Expression summary of Gerbera MADS box genes in different floral organs. The results are from RNA gel blot *and in situ *hybridization data. Click here for file

Additional file 6 **Primer sequences**. Primer sequences of Gerbera MADS box genes used for Gateway (Invitrogen) conversion. Click here for file

Additional file 7 **Alignment of protein sequences**. Alignment of Gerbera GRCD1-5 and Arabidopsis SEP3 protein sequences. Click here for file

Additional file 8 **Yeast three-hybrid analysis**. An example of yeast three-hybrid analysis of MADS-domain proteins on SD -Leu, -Trp, -Ura, -Ade plate. Click here for file

Additional file 9 **Original yeast two-hybrid data**. Scores and interpretation of all yeast two-hybrid data collected. Click here for file
