# Supplementary figures and images for: Large scale interaction analysis predicts that the Gerbera hybrida floral E function is provided both by general and specialized proteins
Source: BMC Plant Biol. 2010 Jun 25;10:129. doi: 10.1186/1471-2229-10-129 (PMC3017775; doi:10.1186/1471-2229-10-129)

**
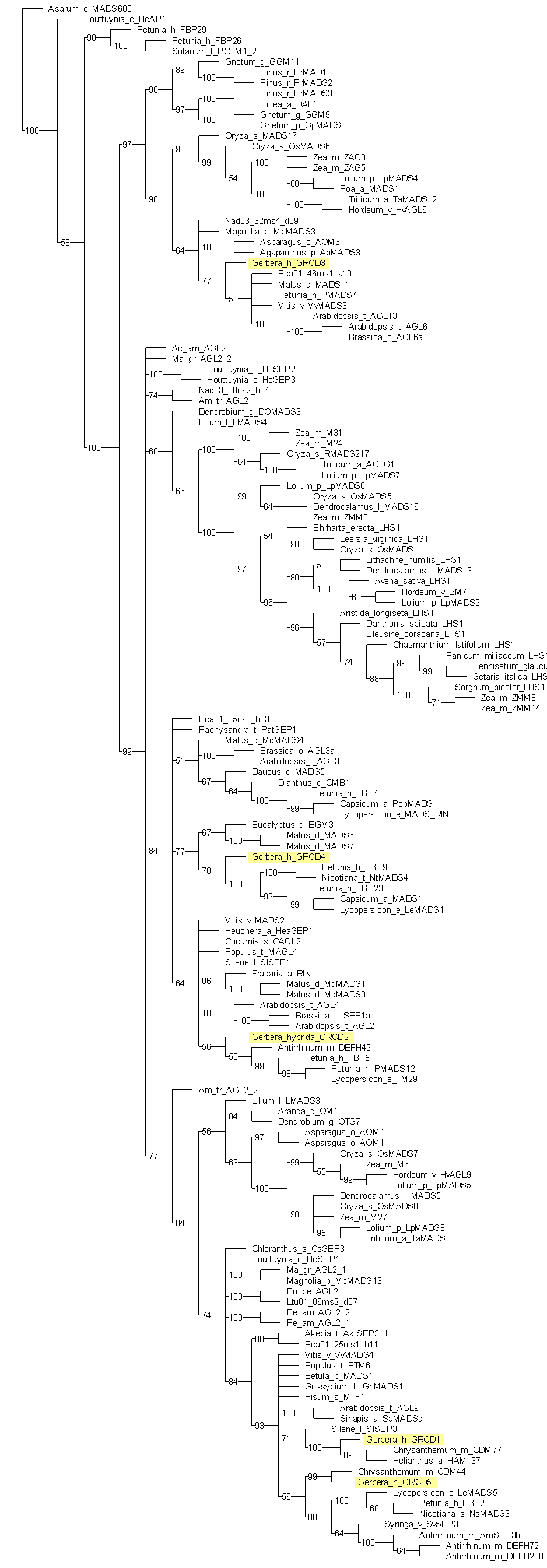
**

**Figure S1**. Phylogenetic tree showing the positions of Gerbera *SEP*-like genes, *GRCD*s.

Supplement: Additional file 1 — Phylogenetic tree. A phylogenetic tree showing the positions of Gerbera SEP-like genes. [file 1471-2229-10-129-S1.DOC]
