# Supplementary material for: Large scale interaction analysis predicts that the Gerbera hybrida floral E function is provided both by general and specialized proteins
Source: BMC Plant Biol. 2010 Jun 25;10:129. doi: 10.1186/1471-2229-10-129 (PMC3017775; doi:10.1186/1471-2229-10-129)
Supplement: Additional file 2 — Gerbera MADS box genes. Summary of Gerbera MADS box genes used in this study. [file 1471-2229-10-129-S2.DOC]

**Table S1**. Summary of Gerbera MADS box genes used in this study.

__________________________________________________________________________

**Gerbera MADS Identified in Source Expression analysis in**

**­box gene**

**__________________________________________________________________________**

***GSQUA1*** Yu *et al.,* 1999 Gerbera inflorescence Yu *et al.,* 1999

[AJ009727] cDNA library

***GSQUA2*** Ruokolainen *et al.,* PCR of Gerbera Ruokolainen *et al.,* 2010

[FN298387] 2010 inflorescence cDNA

***GSQUA3*** Ruokolainen *et al.,* PCR of Gerbera Ruokolainen *et al.,* 2010

[FN298388] 2010 inflorescence cDNA

***GSQUA5*** Laitinen *et al*., 2005 Gerbera EST collection Ruokolainen *et al.,* 2010

[FN298389]

***GGLO1*** Yu *et al*., 1999 Gerbera corolla and Yu *et a*l., 1999

[AJ009726] young inflorescence

cDNA library

***GDEF1*** Yu *et al*., 1999 Gerbera inflorescence Yu *et al*., 1999

[AJ009724] cDNA library

***GDEF2*** Yu *et al*., 1999 Gerbera corolla and Yu *et al*., 1999

[AJ009725] young inflorescence

cDNA library

***GAGA1*** Yu *et al*., 1999 Gerbera inflorescence Yu *et al*., 1999

[AJ009722] cDNA library

***GAGA2*** Yu *et al.,* 1999 Gerbera inflorescence Yu *et al.,* 1999

[AJ009723] cDNA library

***GRCD1*** Kotilainen *et al*., 2000 Gerbera ray flower Kotilainen *et al*., 2000

[AJ400623] petal cDNA library

***GRCD2*** Uimari *et al*., 2004 Gerbera petal cDNA Uimari *et al*., 2004

[AJ784156] library

***GRCD3*** Kotilainen *et al*., 2000 Gerbera cDNA library This study

[AJ784157] Laitinen *et al*., 2005

***GRCD4*** Laitinen *et al*., 2005 Gerbera EST collection This study

[FN297860]

***GRCD5*** Laitinen *et al*., 2005 Gerbera EST collection This study

[FN297861]

**_________________________________________________________________________________________**
