# Supplementary material for: Large scale interaction analysis predicts that the Gerbera hybrida floral E function is provided both by general and specialized proteins
Source: BMC Plant Biol. 2010 Jun 25;10:129. doi: 10.1186/1471-2229-10-129 (PMC3017775; doi:10.1186/1471-2229-10-129)
Supplement: Additional file 3 — Expression during ray flower development. RNA gel blots showing expression of GRCD3, GRCD4 and GRCD5 during Gerbera ray flower development. [file 1471-2229-10-129-S3.PDF]

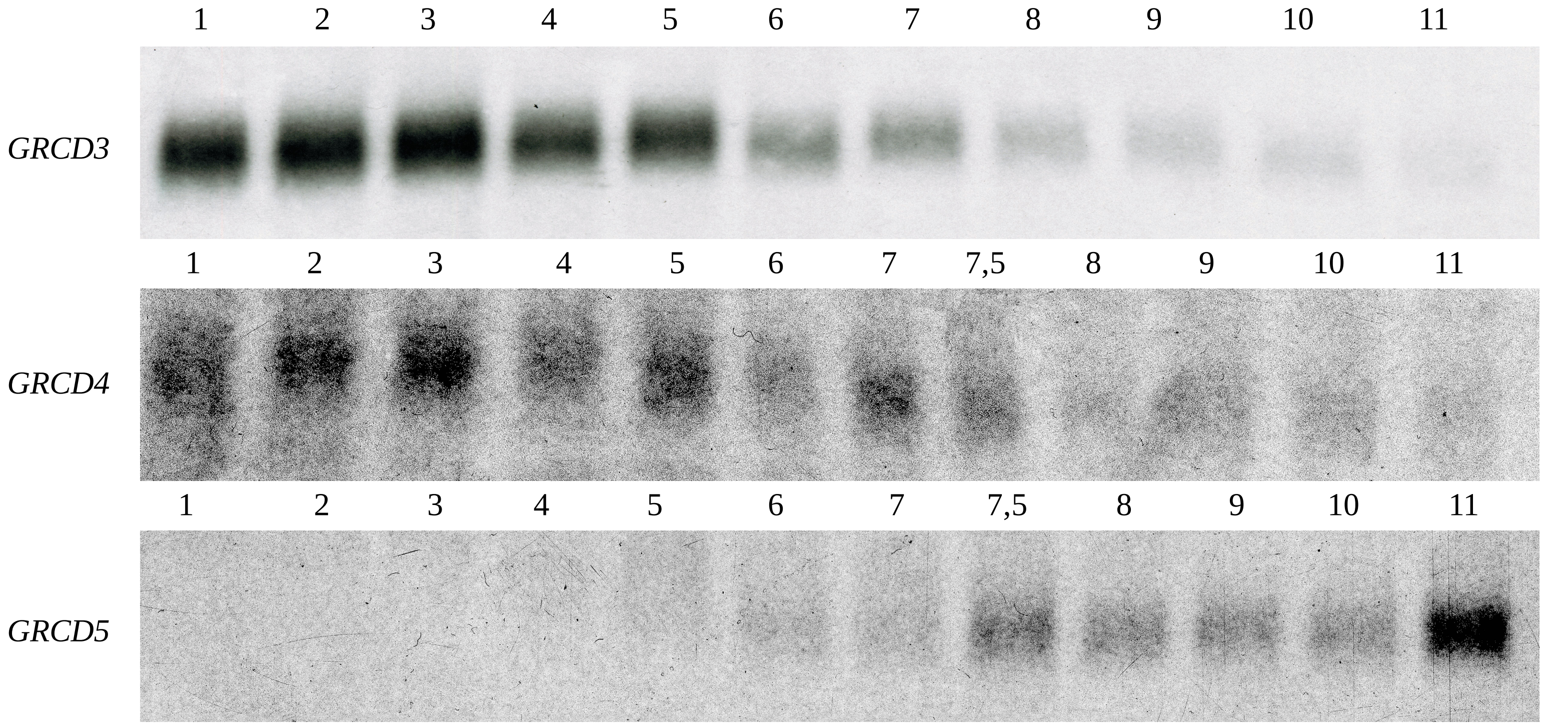

Figure S2. Expression of *GRCD3*, *GRCD4*, and *GRCD5* during Gerbera ray flower petal development. *GRCD3* and *GRCD4* are expressed strongly at the early stages of ray flower petal development. *GRCD5* exhibits an opposite expression pattern by being upregulated at the later stages of petal development. For Gerbera ray flower petal developmental stages see [58].
