# Supplementary material for: Large scale interaction analysis predicts that the Gerbera hybrida floral E function is provided both by general and specialized proteins
Source: BMC Plant Biol. 2010 Jun 25;10:129. doi: 10.1186/1471-2229-10-129 (PMC3017775; doi:10.1186/1471-2229-10-129)
Supplement: Additional file 5 — Expression of Gerbera MADS box genes. Expression summary of Gerbera MADS box genes in different floral organs. The results are from RNA gel blot and in situ hybridization data. [file 1471-2229-10-129-S5.DOC]

**Table S3**. Expression of Gerbera MADS box genes in different floral organs. The results are from RNA gel blot *and in situ* hybridization data (Yu *et al*. 1999, Kotilainen et al. 2000, Uimari *et al.* 2004, Broholm *et al*. 2009, Ruokolainen *et al*. 2010).

|  | W1 | W2 | W3 | W4 | Ovary | Leaf | Root | Vasculature |
| --- | --- | --- | --- | --- | --- | --- | --- | --- |
| *GAGA1* |  |  | x | x | x |  |  |  |
| *GAGA2* |  |  | x | x | x |  |  |  |
| *GGLO1* |  | x | x |  |  |  |  |  |
| *GDEF1* | x | x | x |  | x |  |  |  |
| *GDEF2* |  | x | x |  | x | x |  |  |
| *GRCD1* | x | x | x | x | x |  |  |  |
| *GRCD2* | x | x | x | x | x |  |  |  |
| *GRCD3* | x | x | x | x | x |  |  | x |
| *GRCD4* | x | x | x | x | x |  |  | x |
| *GRCD5* | x | x | x | x | x |  |  | x |
| *GSQUA1* |  | x |  |  |  |  |  | x |
| *GSQUA2* | x | x | x | x | x |  |  | x |
| *GSQUA3* |  | x | x | x | x | x |  | x |
| *GSQUA5* | x | x | x | x | x |  |  | x |
