# Supplementary material for: Large scale interaction analysis predicts that the Gerbera hybrida floral E function is provided both by general and specialized proteins
Source: BMC Plant Biol. 2010 Jun 25;10:129. doi: 10.1186/1471-2229-10-129 (PMC3017775; doi:10.1186/1471-2229-10-129)
Supplement: Additional file 6 — Primer sequences. Primer sequences of Gerbera MADS box genes used for Gateway (Invitrogen) conversion. [file 1471-2229-10-129-S6.DOC]

**Table S4**. Primer sequences of Gerbera MADS box genes used for Gateway (Invitrogen) conversion. The Gateway derived part of primer was added to the 5' end of gene specific primer sequence.

____________________________________________________________________________

**Gene Forward primer 5' Reverse primer 3'**

**____________________________________________________________________________**

***GGLO1*** TCATGGGGAGAGGAAAGATA TTACATCCTCTCATGCAA

***GDEF2*** TCATGGCGAGAGGAAAGATC CTAGCCAAGCAAAGCATA

***GDEF1*** TCATGGGGAGGGGGAAGATA TCAATTAGTGTGTTGATGATCATGGAG

***GAGA1*** TCATGGAAAATTCTGATGTGCTTGAGC TTACACTAACTGGAGCGG

***GAGA2*** CGATGTCGTTTCCAAATGAT TTACACTAATTGGAGAGGT

***GRCD1*** TCATGGGAAAAGGAAGGTTAGAGTT TCATGCTGGCCAACCCTG

***GRCD2*** TCATGGGGAGAGGAAGAGTT TCAAAGCATCCATCCAGG

***GRCD3*** TTATGGGGAGAGGAAGAGTAGAGCT GTCAAAGGACCCAACCATG

***GRCD4*** TCATGGGTAGAGGGAGAG TTATGGATACCAATGGAGTGA

***GRCD5*** CTATGGGGAGAGGAAGAGTAGAA TTAGGGGTTATAGGGAATGGTTTG

***GSQUA1*** TCATGGGTAGAGGAAAGGTACAA TTACCCAATATTGAGAGAAGAATGTGG

***GSQUA2*** TCATGGGAAGAGGGAGGGT CTCATTGATAAATGTGATGAAACAACCA

***GSQUA3*** CCATGGGGAGAGGAAAGGTA TTCATGACGGAAAGCATCT

***GSQUA5*** CTATGGGGAGGGGAAGAGT CTTACTGATTCATGTGCTGAAGCAT

**Gateway** GGGGACAAGTTTGTACAAAA GGGGACCACTTTGTACAAGAAAGCTGGGT

**sequence** AAGCAGGCT

____________________________________________________________________________
