# Supplementary material for: Large scale interaction analysis predicts that the Gerbera hybrida floral E function is provided both by general and specialized proteins
Source: BMC Plant Biol. 2010 Jun 25;10:129. doi: 10.1186/1471-2229-10-129 (PMC3017775; doi:10.1186/1471-2229-10-129)
Supplement: Additional file 7 — Alignment of protein sequences. Alignment of Gerbera GRCD1-5 and Arabidopsis SEP3 protein sequences. [file 1471-2229-10-129-S7.DOC]

GRCD5 **MGRGRVELKRIENKINRQVTFAKRRNGLLKKAYELSVLCDAEVALIIFSNRGKLYEF**CSS

SEP3 **MGRGRVELKRIENKINRQVTFAKRRNGLLKKAYELSVLCDAEVALIIFSNRGKLYEF**CSS

GRCD1 **MGKGRLELKRIENKINRQVTFAKRRNGLLKKAYELSVLCDAEVALIVFSTRGKLYEF**SST

GRCD2 **MGRGRVELKRIENKINRQVTFAKRRNGLLKKAYELSVLCDAEVALIIFSNRGKLFEF**CST

GRCD4 **MGRGRVELKRIENKINRQVTFAKRRNGLLKKAYELSVLCDAEVALIIFSNRGKLYEF**CSS

GRCD3 **MGRGRVELKRIENKINRQVTFSKRRNGLLKKAYELSVLCDAEVGLIIFSSRDKLYEF**GSV

GRCD5 SSMLKTLERYQKCNYGAPDQTNVSAREAL-ELS**SQQEYLKLKARYEALQRSQRNLLGEDL**

SEP3 SSMLRTLERYQKCNYGAPEP-NVPSREALAELS**SQQEYLKLKERYDALQRTQRNLLGEDL**

GRCD1 SSMLKTLERYEKCSFGPPEQRRPAAKEDLQEQS**SYQEYMRLKERYDALKRLERNYYGEEI**

GRCD2 SNMLKMLERYQNCTYG---SMEVDRSTPNAEQS**SYKEYMKLKAKYESLQQYQRQLFGEDL**

GRCD4 SSMVKTLEKYHSCSYG---SLKASQPE-NESQY**NYHEYLRLKARVEVLQRSQRNLLGEDL**

GRCD3 G-VMKTLERYQRCCFN-----PQDNNNERETQS**WYQEVSKLKAKFESLQRTQRHLLGEDL**

GRCD5 **GPLNCKELESLERQLDTSLKHIRSARTQLMLDTLTDLQKKEHALNE**ANRTLKQRLIEGTQ

SEP3 **GPLSTKELESLERQLDSSLKQIRALRTQFMLDQLND**L**QSKERMLTE**TNKTLRLRLADGYQ

GRCD1 **DSLTTSELESLERQLHCSLKQIRTIRTQSLLDKLYEQQKMEHQLYE**SNKTLRLRLDEEGQ

GRCD2 **GPLSLKELEQLERQLDSTLRQIRSIRTQSMLDRLSELQVKERMWVE**ANKALQNKLEEVYA

GRCD4 **APLNTKELEQLEHQLEMSLRKIRSTKTQSMLDQLADLQRKEQVLAE**TNKALRKKLEESAQ

GRCD3 **GPLSVKELHNLEKQLEGALTQARQRKTQIMVEQMEELRRKERELGD**MNKHLKIKVSHELS

GRCD5 INSLHWYP**QAPQEVCYDDRQHAPQHQTDEAFFHPLDCG--PTLQIGYQ--------TDPI**

SEP3 MP-LQLNPNQ-EEVDHYGRHHHQQQQHSQAFFQPLECE--PILQIGYQ--------GQQD

GRCD1 AEALQWDAHAHANGMVYAHQHQVSQPMRDTFYHPTGCE--TTLQIGYQ--------SEQ-

GRCD2 ENQAGPSWAAGEHHSSYGQEHQHQHQS-QGFFQPLDCN--SNLQIGYN------TVDSSH

GRCD4 EYPVRQMWEGGAQTIPY---NP**LPTHS-DEFFQPLGLN--STMHNSFNGLRYNPIVSDEM**

GRCD3 TFDAEGQGYRAQLPCPWNSGTNNTFTMHPSQSNPMDCQQEPILQIGYN----QFMHGEGS

GRCD5 **NVAGAEAVAGPSMNNYMQGWLPC-----**

SEP3 GMG-----AGPSVNNYMLGWLPYDTNSI

GRCD1 ---------MSAVNHQMQGWPA------

GRCD2 ITAS---TNGQNLNGLIPGWML------

GRCD4 **NVAG---ANNNSPNGLFPGWML------**

GRCD3 SVQR-----NMVGENGIHGWVL------

**Figure S3.** Alignment of Gerbera GRCD1-5 and Arabidopsis SEP3 protein sequences. The MADS domain is shown in red and the K domain is shown in blue. The C-terminal sequence for GRCD4 and GRCD5 shown in green was deleted for protein-protein interaction studies due to strong autoactivation.
