# Supplementary material for: Large scale interaction analysis predicts that the Gerbera hybrida floral E function is provided both by general and specialized proteins
Source: BMC Plant Biol. 2010 Jun 25;10:129. doi: 10.1186/1471-2229-10-129 (PMC3017775; doi:10.1186/1471-2229-10-129)
Supplement: Additional file 8 — Yeast three-hybrid analysis. An example of yeast three-hybrid analysis of MADS-domain proteins on SD -Leu, -Trp, -Ura, -Ade plate. [file 1471-2229-10-129-S8.DOC]

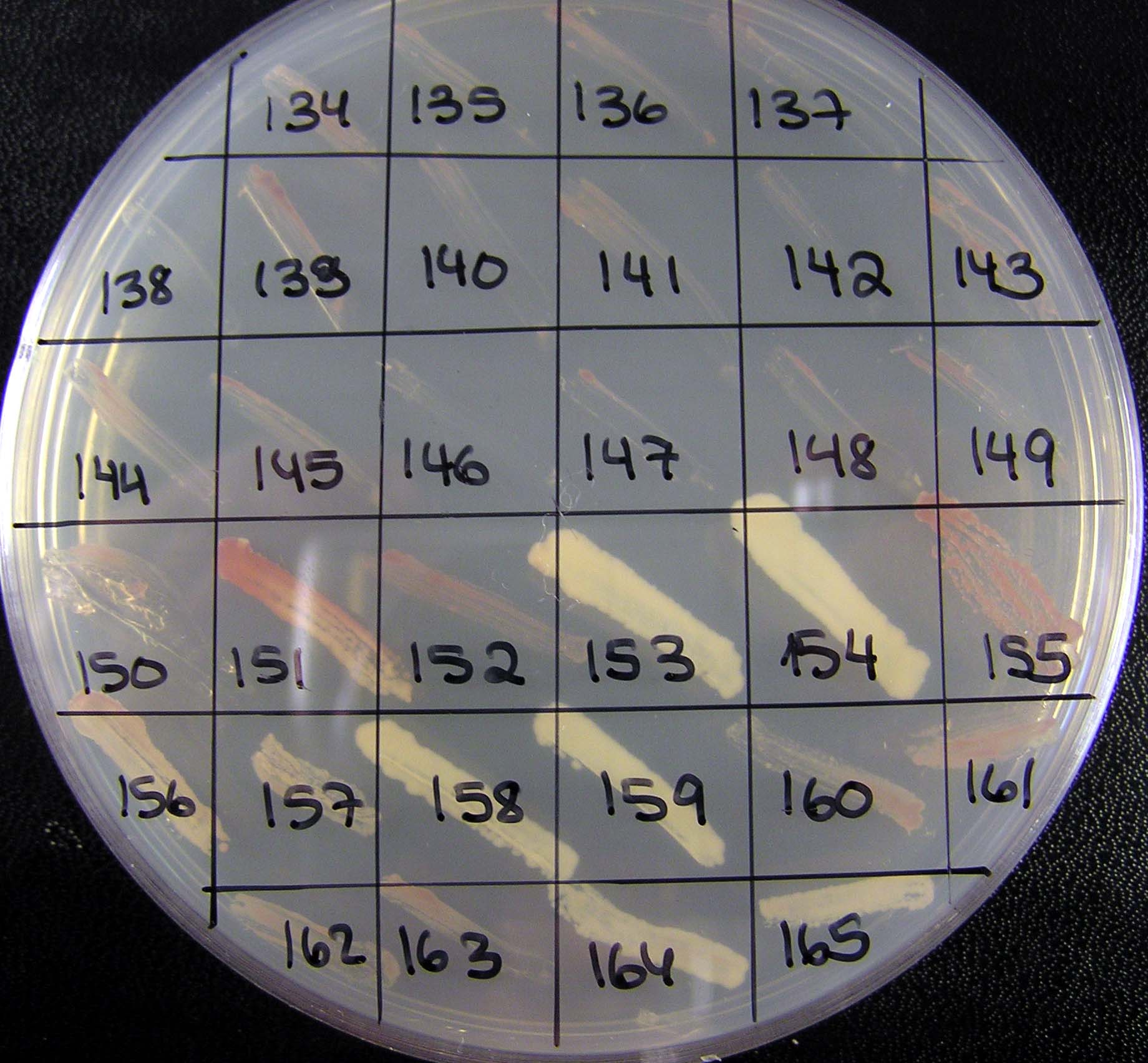


**Figure S4**. Yeast three-hybrid analysis on SD -Leu, -Trp, -Ura, -Ade plate. The numbers refer to the combinations of different Gerbera MADS domain proteins depicted in the Supplemental Table 4.
